# Colonic Mucosa-Associated Lymphoid Tissue Following Prior Hodgkin Lymphoma: A Diagnostic Challenge and Therapeutic Approach – A Case Report

**DOI:** 10.1159/pjg/acwag011

**Published:** 2026-10-15

**Authors:** Beatriz Pereira, Rui Mendo, Bárbara Pena, Filipa Campos, Catarina Vieira

**Affiliations:** a Serviço de Gastrenterologia, Unidade Local de Saúde de Lisboa Ocidental, Lisbon, Portugal; b Serviço de Anatomia Patológica, Unidade Local de Saúde Arco Ribeirinho, Barreiro, Portugal; c Serviço de Hematologia, Unidade Local de Saúde Almada-Seixal, Almada, Portugal; d Serviço de Gastrenterologia, Unidade Local de Saúde Arco Ribeirinho, Barreiro, Portugal

**Keywords:** Mucosa-associated lymphoid tissue lymphoma, Colorectal lymphoma, Marginal zone lymphoma, Endoscopic mucosal resection, Rituximab, Hodgkin lymphoma

## Abstract

**Introduction:**

Colorectal mucosa-associated lymphoid tissue (MALT) lymphoma is a rare entity, accounting for approximately 1.6% of all MALT lymphoma cases. Its clinical and endoscopic presentation is generally nonspecific, and given the limited number of reported cases, no consensus exists on the optimal therapeutic strategy.

**Case Presentation:**

A 47-year-old male with a 15-year remission history of stage IIb nodular lymphocyte-predominant Hodgkin lymphoma (NLPHL) underwent abdominopelvic CT for recurrent urinary tract infections, revealing a suspected sigmoid neoplasm with locoregional lymphadenopathy. Colonoscopy showed two adjacent but noncontiguous subepithelial erythematous lesions (15 mm and 10 mm) with surrounding “chicken skin” mucosal pattern. Initial biopsies demonstrated chronic inflammation only. At endoscopic reassessment, endoscopic mucosal resection (EMR) was performed, yielding a histological diagnosis of MALT lymphoma. Following multidisciplinary discussion, the patient underwent four cycles of rituximab monotherapy, achieving complete remission on reassessment CT. At eighteen months of follow-up, he remains asymptomatic without clinical, radiological, or endoscopic evidence of relapse.

**Conclusion:**

We report the first described coexistence of colonic MALT lymphoma and prior NLPHL, without an established biological association given the absence of comparative molecular studies. This case highlights the role of EMR when conventional biopsies are nondiagnostic and demonstrates the efficacy of rituximab monotherapy in this rare entity.

## Introduction

Mucosa-associated lymphoid tissue (MALT) lymphoma is an indolent B-cell non-Hodgkin lymphoma (NHL) arising from the malignant transformation of B cells at extranodal sites. Although predominant in the gastrointestinal tract, colorectal MALT lymphoma accounts for approximately 1.6% of all MALT lymphomas, with a limited number of cases reported in the literature [1]. No guidelines exist regarding the best therapeutic strategy, and an individualized approach is recommended.

Herein, we describe a case of sigmoid MALT lymphoma incidentally detected on cross-sectional imaging in a patient with a prior history of Hodgkin lymphoma (HL). This case emphasizes the diagnostic challenge posed by nondiagnostic initial forceps biopsies and the critical role of endoscopic mucosal resection (EMR) in obtaining adequate tissue for definitive diagnosis and guiding therapy.

## Case Presentation

We present the case of a 47-year-old Caucasian male with a relevant medical history of stage IIb nodular lymphocyte-predominant Hodgkin lymphoma (NLPHL), diagnosed 20 years earlier; details of the initial treatment regimen are not available. He experienced relapse 15 years ago, treated with chlorambucil, vinblastine, procarbazine, and prednisolone chemotherapy, achieving sustained remission to date. There was no known family history of hematological or gastrointestinal malignancies, no regular medication use, and no history of smoking, alcohol abuse, or recreational drug use. He underwent abdominopelvic CT (computerized tomography) for recurrent urinary tract infections and denied any complaints of abdominal pain, gastrointestinal bleeding, or changes in bowel habits. Imaging demonstrated a sigmoid colon lesion with locoregional lymphadenopathy, raising suspicion for a neoplastic process, without evidence of upstream mechanical obstruction (shown in Fig. 1).

**Fig. 1.**
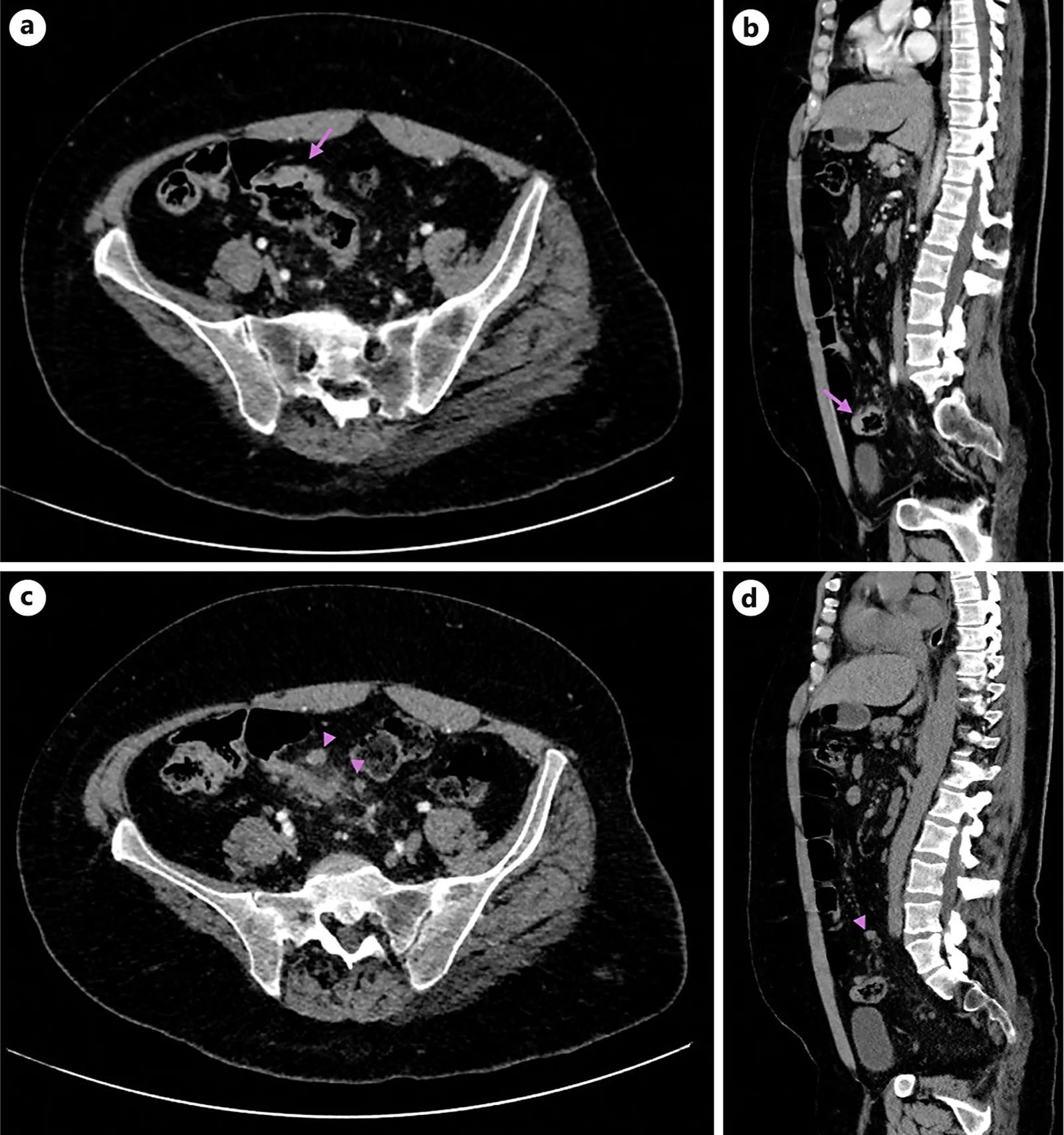
Abdominopelvic CT scan – axial (panel **a** and **c**) and sagittal (panel **b** and **d**) sections – showing eccentric parietal thickening of the sigmoid colon (arrows) with infiltrative densification of the adjacent fat with locoregional lymphadenopathy (arrowheads).

Subsequent colonoscopy revealed two adjacent but noncontiguous erythematous lesions in the distal sigmoid colon (15 mm and 10 mm), 20 cm from the anal verge, with the surface showing superficial erosions and adherent exudate and the surrounding mucosa displaying a “chicken skin” appearance (shown in Fig. 2). No synchronous lesions were identified in the remaining colonic segments. Initial tunnel biopsies identified chronic inflammation without dysplasia or malignancy. Physical examination was unremarkable. Routine blood counts were normal, biochemical tests showed no significant abnormalities, and serologies were negative for hepatitis B, hepatitis C, and human imunodeficiency virus.

**Fig. 2.**
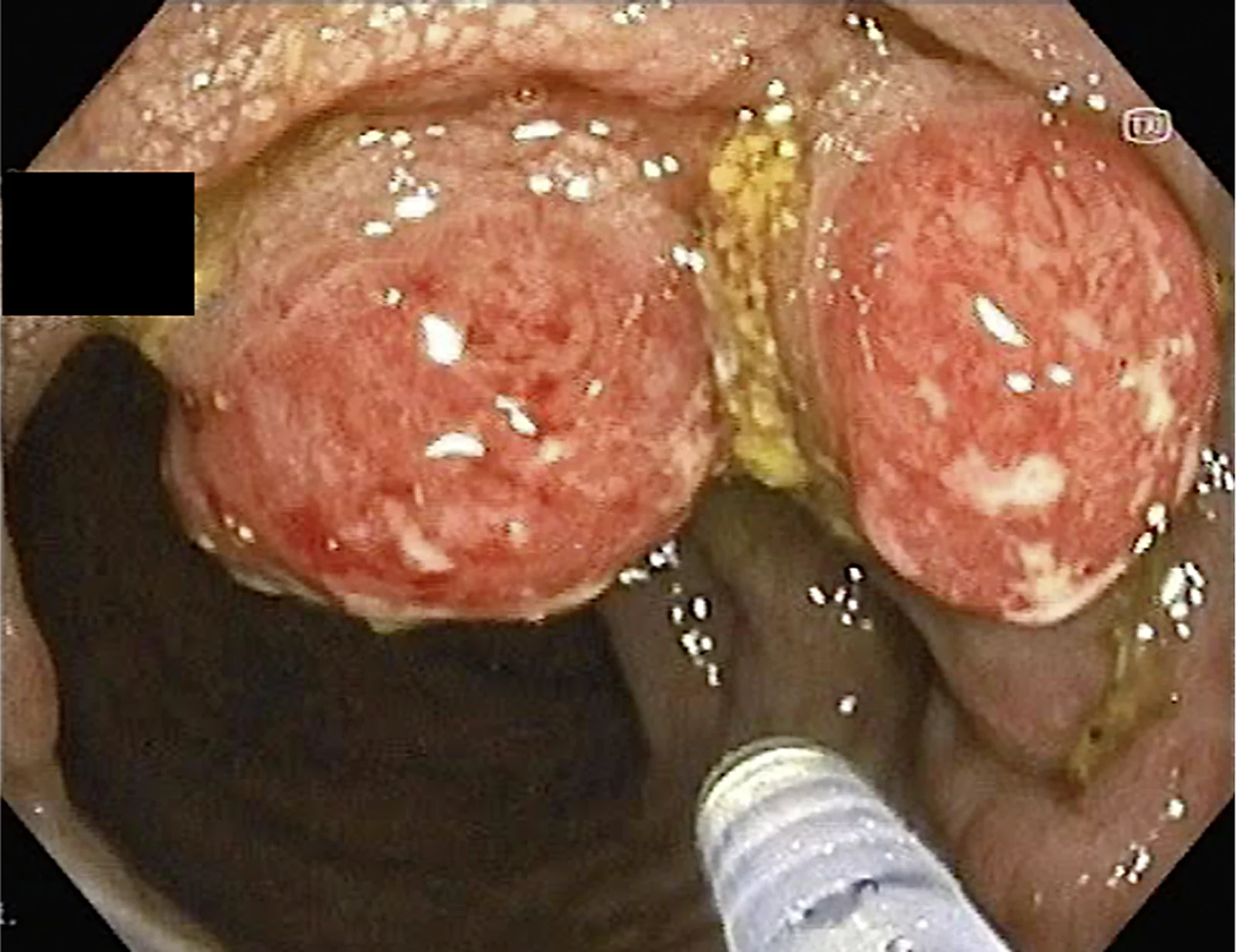
Endoscopic appearance of the two adjacent but noncontiguous erythematous subepithelial lesions (15 mm and 10 mm) in the sigmoid colon. Surrounding mucosa displays the characteristic “chicken skin” pattern.

Given the diagnostic discordance between imaging, endoscopic appearance, and initial biopsy results, a repeat colonoscopy with EMR of both lesions was performed to achieve a histopathologic diagnosis. Submucosal injection using a normal saline, adrenaline, and methylene blue solution achieved a satisfactory lifting effect. En bloc mucosectomy was successfully performed. Superficial involvement of the muscularis propria was noted at the resection base of the larger lesion (Sydney type 2 defect), prompting prophylactic closure with four endoscopic clips. No other complications were observed.

Macroscopic evaluation revealed markedly firm lesions. Histological examination demonstrated fragments of distal intestinal mucosa without dysplasia, with submucosal infiltration by small lymphoid cells with an immunophenotype consistent with MALT lymphoma: CD20 and BCL-2 positive, focal CD5/CD43 positivity, negative for CD3, CD10, BCL-6, and cyclin D1, with a low proliferative index of approximately 5% (shown in Fig. 3). No evidence of high-grade transformation was identified. The patient was referred to hematology and, following multidisciplinary discussion, underwent four cycles of rituximab monotherapy, administered as a standard 4-week induction regimen (one weekly cycle), well tolerated without adverse events. A whole-body CT three months after the final cycle showed no evidence of residual or extra-colonic disease, with complete resolution of the previously described lymphadenopathies. Surveillance colonoscopy performed after completion of rituximab demonstrated complete endoscopic resolution of the MALT lymphoma, with no residual lesions at the prior resection sites (shown in Fig. 4). Biopsies of the scar were not obtained, representing a limitation of posttreatment confirmation in this case. At 18 months of follow-up, the patient remains asymptomatic with no clinical, radiological, or endoscopic evidence of relapse.

**Fig. 3.**
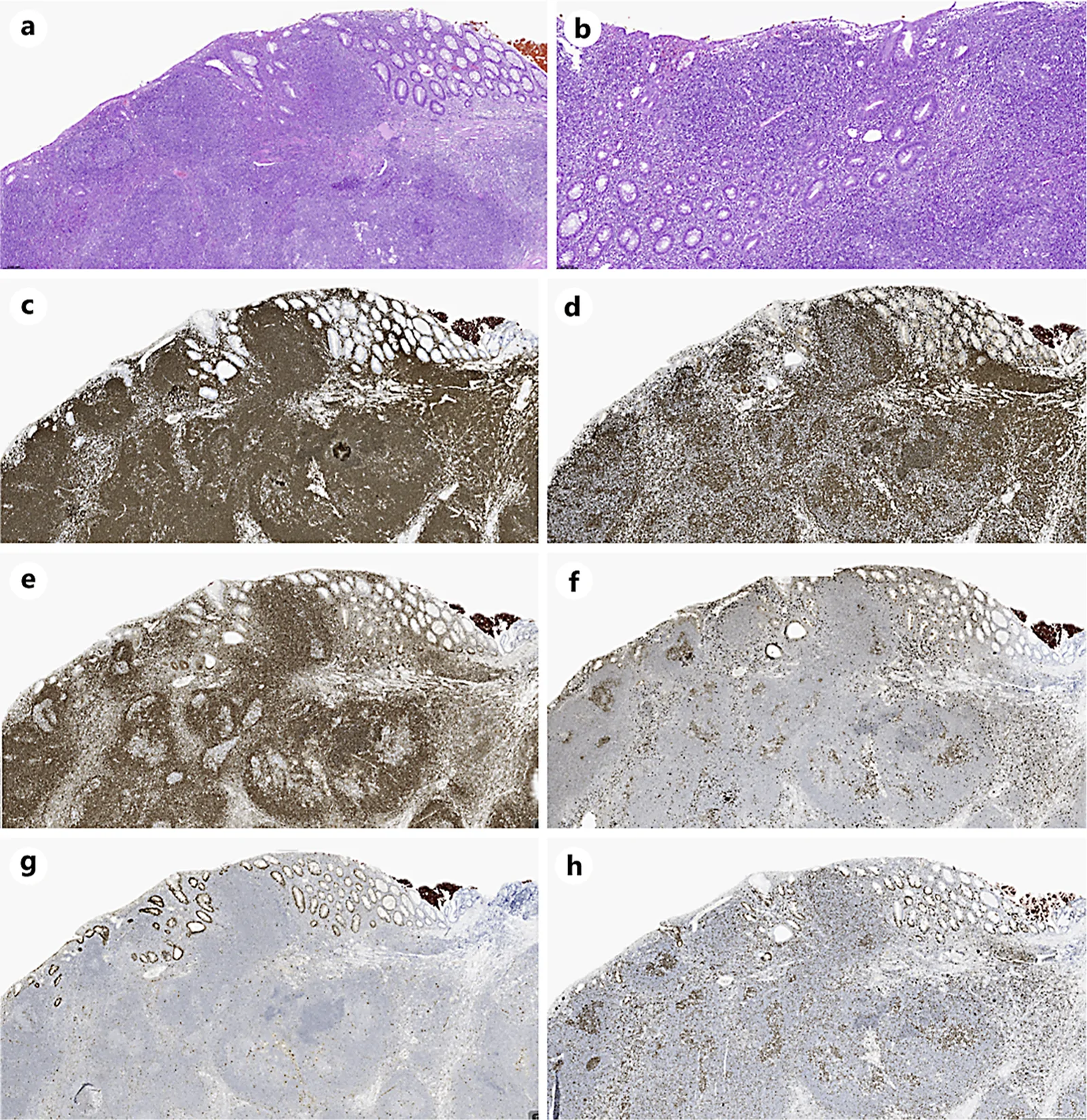
Histological section of mucosa from the distal portion of the large intestine showing submucosal infiltration by small lymphoid cells compatible with MALT lymphoma. Hematoxylin and eosin staining, original magnification ×5 (panel **a**) and ×10 (panel **b**). Immunohistochemical profile: CD20+ (panel **c**), CD5+ (panel **d**), BCL-2+ (panel **e**), CD10− (panel **f**), cyclin D1− (panel **g**); Ki-67 of 5% (panel **h**). No evidence of high-grade transformation.

**Fig. 4.**
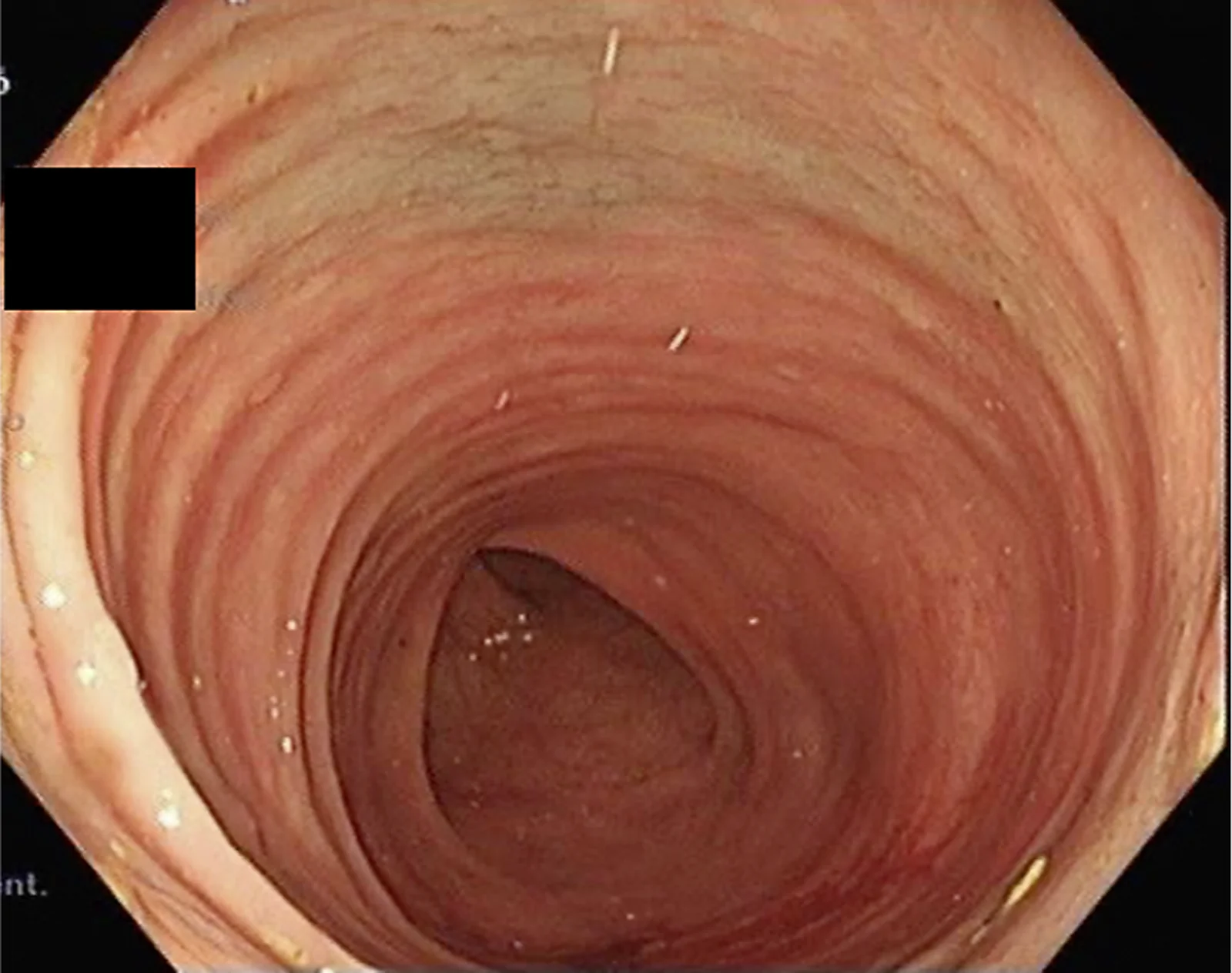
Follow-up colonoscopy performed after rituximab completion and EMR confirmed with no evidence of residual disease at the previously affected site.

## Discussion

Marginal zone B-cell lymphoma (MZL) encompasses a group of indolent B-cell neoplasms and is formally categorized into extranodal MZL of the MALT (also known as MALT lymphoma), splenic MZL, and nodal MZL [2, 3]. MALT lymphoma predominantly affects the stomach. Colorectal involvement accounts for only 1.6% of the cases, most frequently in the rectum (39.2%), with sigmoid localization in just 2% of cases, and multisegmental disease in 15–20% [4, 5]. The mean age at diagnosis is 60 years, with a similar prevalence between genders [4–7].

Chronic antigenic stimulation is central to the pathogenesis of MALT lymphoma. *H pylori* is the most well-established predisposing factor, implicated in approximately two-thirds of gastric MALT cases [8, 9]. In colonic MALT lymphoma, *H pylori* prevalence is lower (∼20%) and its pathogenic role remains unclear [10]. Genetic mechanisms appear more prominent at this site, with chromosomal translocations converging on constitutive NF-κB activation and TNFAIP3 (A20) inactivation contributing to neoplastic transformation independent of external antigenic drive [11].

Colonic MALT lymphomas are typically localized and slow-growing; most cases are asymptomatic and incidentally detected during imaging or screening colonoscopy – as in the present case [5]. When symptomatic, gastrointestinal bleeding, abdominal pain, and altered bowel habits predominate [5–7, 12]. Endoscopically, lesions can be present as polypoid (isolated or multiple small polypoid lesions), mass-forming (protruding lesions with redness, vascularity, or occasionally erosion or ulceration on surface), subepithelial (flat, laterally spreading lesions with smooth surfaces), or inflammatory (mucosal erythema, erosions, or ulceration not fitting the other types) patterns, with flat lesions particularly prone to being missed on standard white-light endoscopy [4, 13]. Given this morphological diversity and its resemblance to adenocarcinoma or other benign conditions, endoscopic diagnosis of colonic MALT lymphoma remains particularly challenging [6].

Definitive diagnosis requires biopsy with integration of histopathological, immunophenotypic, and genetic findings. Histologically, MALT lymphoma is composed of small B cells that typically include marginal zone cells (small- to intermediate-sized cells with pale cytoplasm and slightly irregular nuclei), monocytoid cells, and small lymphocytes, with scattered immunoblasts and centroblast-like cells; lymphoma cells typically infiltrate the epithelium, forming lymphoepithelial lesions, and develop against a background of chronic inflammation that may be difficult to distinguish from lymphoma tissue without adequate tissue sampling [1, 13–15]. Routine forceps biopsies establish the diagnosis in approximately 80% of cases. Occasionally, EMR or endoscopic submucosal dissection (ESD) is needed for the diagnosis and/or local treatment [6, 7]. ESD allows en bloc excision even with large lesions where EMR is not feasible, significantly improving diagnostic yield [6]. The immunophenotype mirrors that of normal marginal zone B cells: CD20+, BCL-2+, BCL-6−, CD5−, CD10−, CD23−, CD43+/−, with a low proliferation rate (Ki-67 <5%). As there is no specific marker for MALT lymphoma, immunohistochemical exclusion of mantle cell lymphoma (CD5, cyclin D1) and follicular lymphoma (CD10, BCL-6) is required – all negative in the present case [16].

Staging workup includes laboratory tests, upper and lower endoscopy, and CT or PET-CT of the thorax, abdomen, and pelvis, using the Lugano modification of Ann Arbor staging [16, 17]. In this case, CT and colonoscopy findings were consistent with stage IIE disease. The clinical benefit of FDG-PET for the staging of patients with indolent lymphomas is not well stablished, given that FDG uptake in MALT lymphoma is variable [18]. Therefore, PET was not performed in this case given the localized disease, low-grade histology, low proliferative index, and absence of high-grade transformation. Despite en bloc EMR, margin status could not be formally assessed due to fragmentation of the resected specimens. Current European Society for Medical Oncology guidelines do not routinely recommend PET in this setting [19]. The absence of upper endoscopy with gastric biopsies and *H pylori* testing represents a recognized limitation of the staging and etiological workup. Although *H pylori* prevalence in colonic MALT is lower than in gastric disease, synchronous gastric involvement would have modified staging, and its systematic exclusion should be incorporated into the workup of future cases.

No standardized treatment protocol exists for colorectal MALT lymphoma, and an individualized approach is recommended [17]. For localized disease, options include endoscopic or surgical resection, radiotherapy, systemic therapy, or active surveillance. Endoscopic mucosal resection is effective for superficial lesions, with ESD preferred for larger lesions or as salvage for residual or recurrent disease [14, 20]. For residual disease or positive margins, involved-site radiotherapy or systemic therapy may be considered; extensive surgery should be reserved for complications [4, 7, 20]. Rituximab monotherapy, extrapolated from nongastric MALT data, has achieved remission in localized cases but persistent disease has also been reported [5, 21]. In this particular case, EMR allowed for an accurate diagnosis and rituximab allowed for the control of both residual local and systemic disease controls, while avoiding the toxicity of full chemoimmunotherapy – resulting in complete remission at 18 months [15, 16]. R-CHOP is generally reserved for confirmed histological transformation to diffuse large B-cell lymphoma [16, 17]. The 18-month follow-up period remains insufficient to establish durable remission in an indolent lymphoma whose natural history and relapse risk are measured in years, and this should be regarded as a limitation of the present report.

Colorectal MALT lymphoma carries a favorable prognosis, with a recent multicenter study reporting 5-year overall survival of 98% and disease-specific survival of 100% at 5 years, reflecting the indolent nature of the disease [4]. Early diagnosis enabling less invasive therapy and the absence of aggressive histological transformation account for similar outcomes across treatment modalities in early-stage disease [4, 6, 22].

The relationship between the patient’s prior HL and subsequent development of MALT lymphoma warrants consideration. To the authors’ knowledge, this is one of the very few reported cases and possibly the first reported coexistence of colonic MALT lymphoma and prior HL. This relationship is particularly relevant in the setting of NLPHL, which is biologically a B-cell neoplasm derived from germinal center B cells and has been reclassified as nodular lymphocyte-predominant B-cell lymphoma in the 5th edition of the WHO classification [2]. Patients with NLPHL are known to have an increased long-term risk of subsequent NHLs, either through histological transformation or through the development of secondary clonally distinct lymphoid neoplasms. In more than half of cases of consecutive HL and NHL, both lymphomas appear to share a common clonal germinal center B-cell precursor; when clonally unrelated, the second lymphoma may represent an independent lymphoid neoplasm potentially facilitated by treatment-related immune dysregulation, chronic antigenic stimulation, host susceptibility factors, or prior chemotherapy-related immune remodeling. [23]. In the present case, comparative molecular characterization – including comparative immunoglobulin gene rearrangement studies and FISH analysis performed after microdissection of tumor cells from both lymphomas – was precluded due to the unavailability of archival tissue from the index HL diagnosis, made 15 years earlier, and a clonal relationship could not be confirmed or excluded. Therefore, both hypotheses – a shared precursor origin or an independent secondary lymphoproliferative disorder – remain biologically plausible [23].

This case illustrates that colorectal MALT lymphoma can present as multifocal subepithelial lesions with nonspecific endoscopic features; and that EMR is valuable when conventional biopsies fail to establish a diagnosis, to obtain adequate deeper tissue for definitive histopathological characterization, while also representing a minimally invasive strategy potentially contributing to local disease control. From a clinical and hematological perspective, relapse or histological transformation would be the leading diagnostic consideration in a patient with prior NLPHL presenting with new lymphoid lesions and adenopathy. However, the final diagnosis of colorectal MALT lymphoma underscores the importance of not assuming biological continuity between sequential lymphoid proliferations without definitive pathologic characterization. A multidisciplinary and individualized approach was crucial in guiding optimal treatment for a condition with sparse available evidence.

We used the CARE reporting guideline to draft this manuscript, and the CARE reporting checklist [24] used when editing, is included in online supplementary material (for all online suppl. material, see https://doi.org/10.1159/pjg/acwag011).

## Statement of Ethics

Written informed consent was obtained from the patient for publication of this case report and any accompanying images. This type of manuscript does not require an ethical approval according to national laws.

## Conflict of Interest Statement

The authors have no conflicts of interest to declare.

## Funding Sources

This study was not supported by any sponsor or funder.

## Author Contributions

Beatriz Pereira was responsible for conception of the case report, literature review, and drafting of the manuscript. Rui Mendo contributed to critical revision of the manuscript for important intellectual content and provided clinical input. Bárbara Pena was responsible for pathological image acquisition and description of histopathological findings. Filipa Campos contributed to clinical interpretation, manuscript revision, and hematological input. Catarina Vieira contributed to final approval of the version to be published. All authors reviewed and approved the final manuscript.

## Data Availability

All data regarding this clinical case study are included in this article and its online supplementary material. Further inquiries may be directed to the corresponding author.
